# Supplementary material for: Tuning cyanide coordination electronic structure enables stable Prussian blue analogues for sodium-ion batteries
Source: Nat Commun. 2025 Nov 18;16:10083. doi: 10.1038/s41467-025-65062-x (PMC12627476; doi:10.1038/s41467-025-65062-x)
Supplement: Supplementary file 1 — Supplementary Information [file 41467_2025_65062_MOESM1_ESM.pdf]

Supplementary Information

Tuning Cyanide Coordination Electronic Structure Enables Stable Prussian Blue Analogues for Sodium-ion Batteries

Yuanheng Wang [1], Jiaxin Yan [1], Bingxing Xie [2,*], Yan Meng [1], Chuankai Fu [1], Fanpeng Kong [1], Xingyu Wang [1], Qingjie Zhou [1], Xin Chen [1], Jianting Li [1], Chunyu Du [1], Liguang Wang [3,*], Pengjian Zuo [1,*]

[1] State Key Laboratory of Space Power-Sources, MIIT Key Laboratory of Critical Materials Technology for New Energy Conversion and Storage, School of Chemistry and Chemical Engineering, Harbin Institute of Technology, No.92 West-Da Zhi Street, Harbin 150001, China

[2] School of New Energy, Nanjing University of Science and Technology, Jiangyin 214443, PR China

[3] College of Chemical and Biological Engineering, Zhejiang University, Hangzhou, 310058, China

Corresponding Author

*zuopj@hit.edu.cn; bingxingxie@njust.edu.cn; wanglg@zju.edu.cn

Supporting equation

$$i = av^b \qquad \text{Equation S1}$$

$$\log(i) = b\log(v) + \log(a) \qquad \text{Equation S2}$$

where i and v represent the peak current and scan rate, respectively, a and b are constant. The value of b reveals the sodium storage mechanism, i.e., a diffusion-limited process with $b = 0.5$, a surface-limited process with $b = 1.0$.

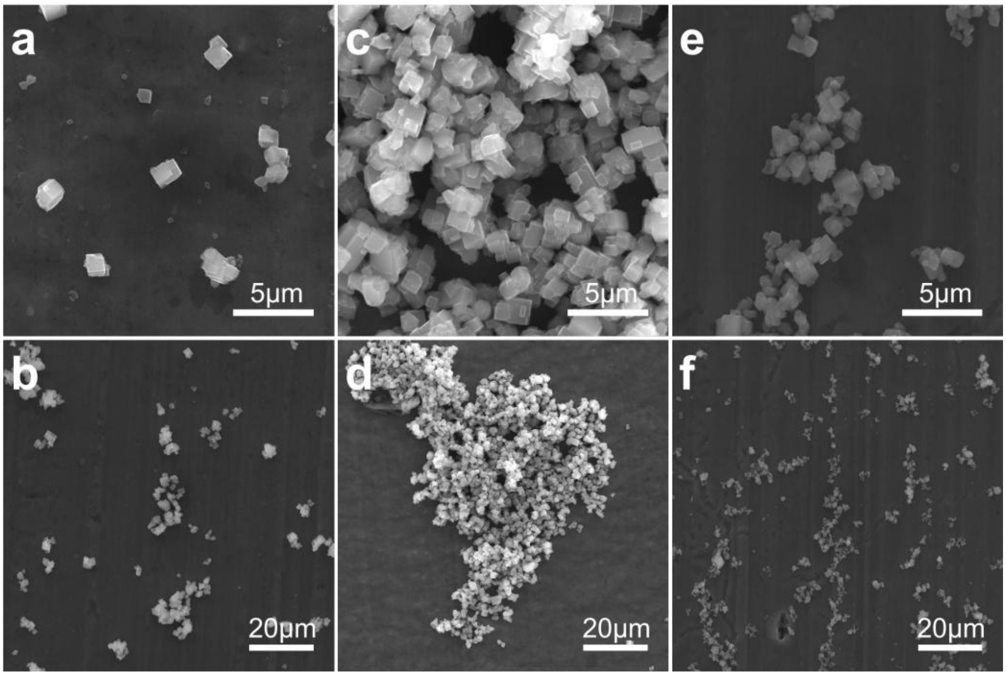

Fig. S1 SEM images of the samples: **a**, **b** M2-PBA, **c**, **d** M4-PBA, and **e**, **f** M5-PBA

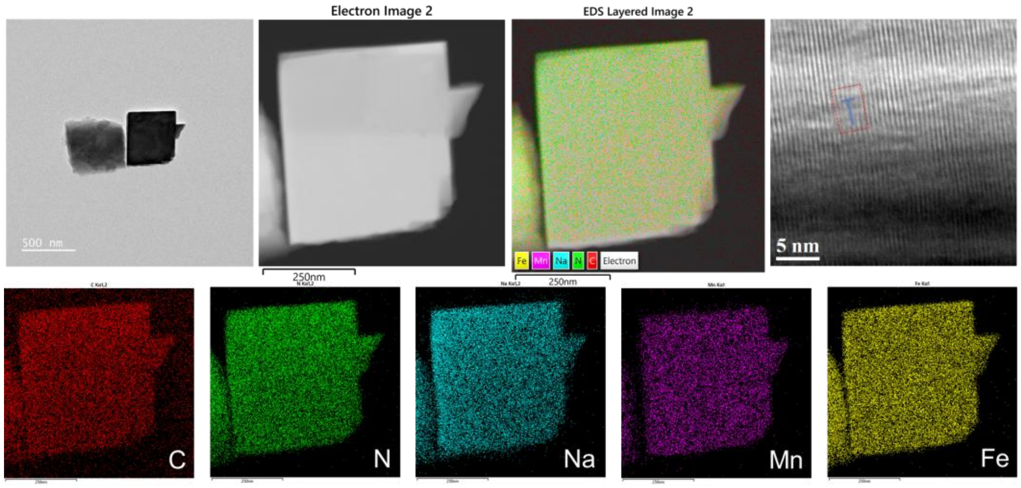

Fig. S2 TEM images of M2-PBA

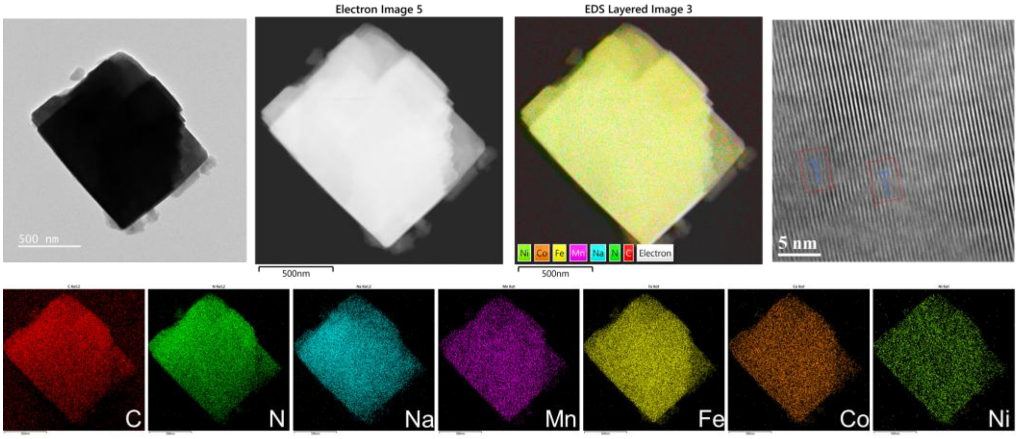

Fig. S3 TEM images of M4-PBA

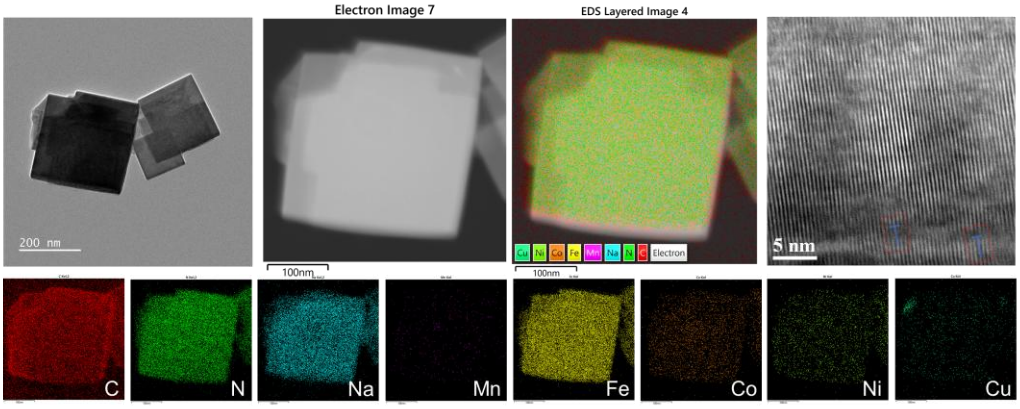

Fig. S4 TEM images of M5-PBA

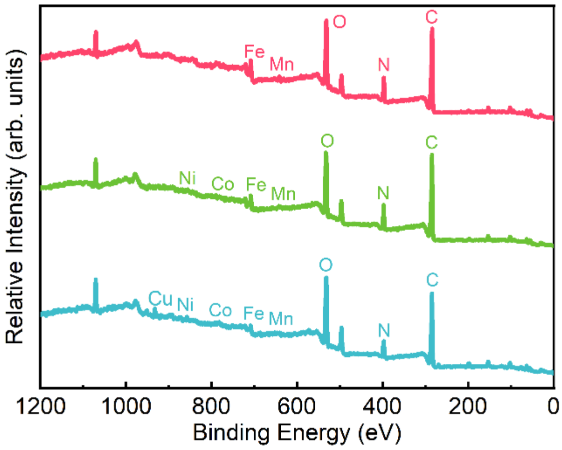

Fig. S5 XPS full spectrum of the three samples with M2-PBA in red, M4-PBA in green, M5-PBA in blue.

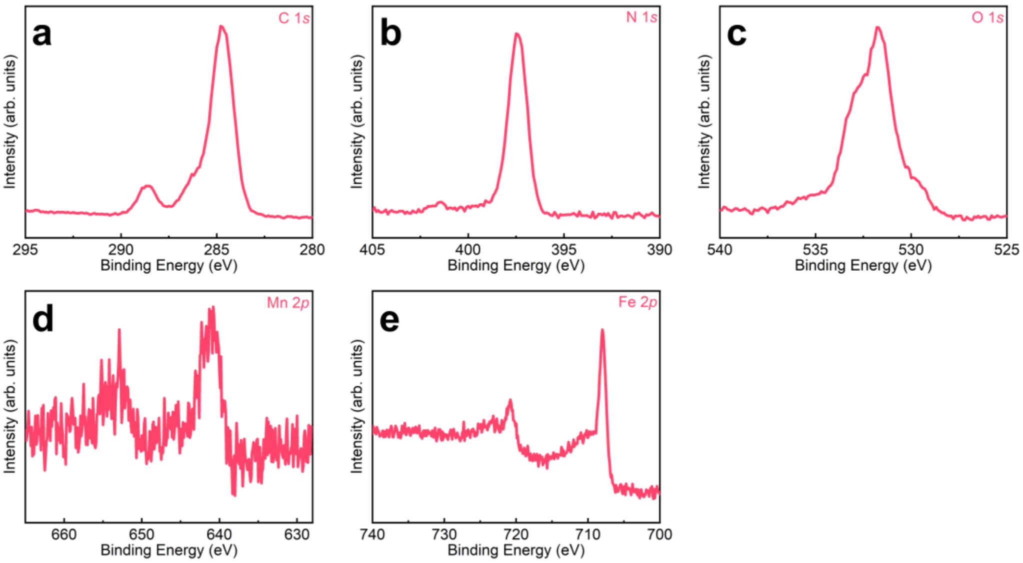

Fig. S6 XPS fine spectrum of M2-PBA. **a** C 1*s*, **b** N 1*s*, **c** O 1*s*, **d** Mn 2*p*, and **e** Fe 2*p*.

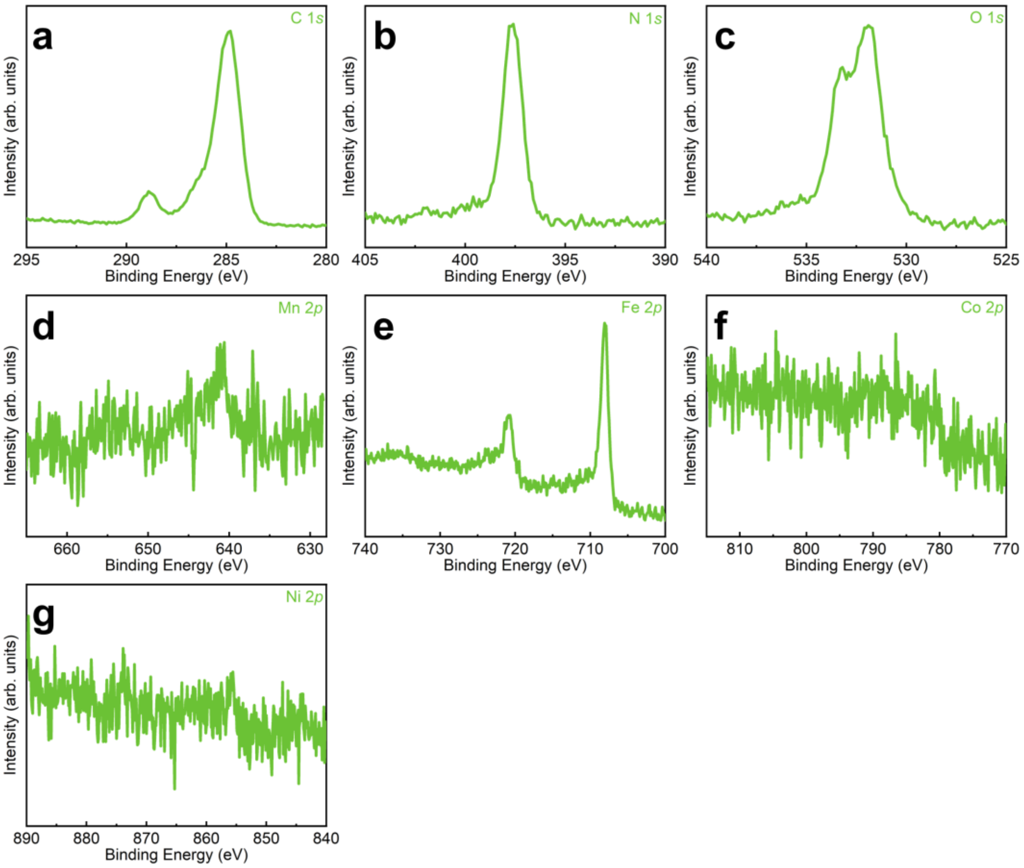

Fig. S7 XPS fine spectrum of M4-PBA. **a** C 1*s*, **b** N 1*s*, **c** O 1*s*, **d** Mn 2*p*, **e** Fe 2*p*, **f** Co 2*p*, and **g** Ni 2*p*.

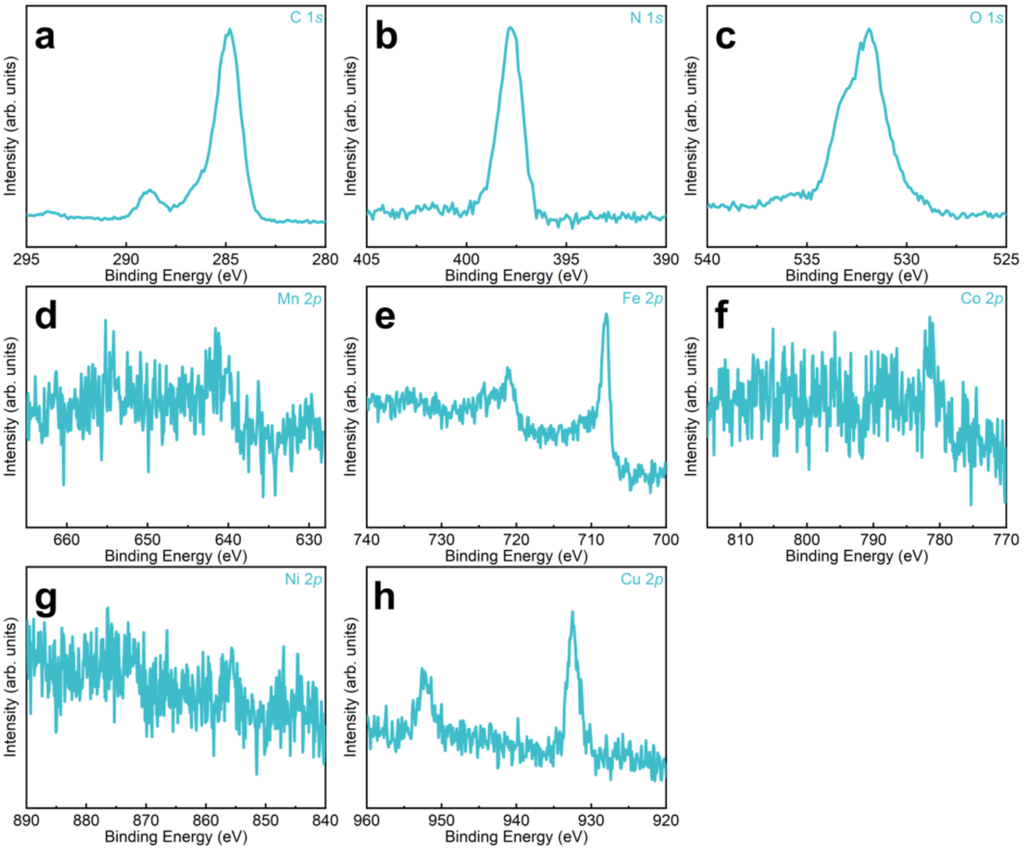

Fig. S8 XPS fine spectrum of M5-PBA. **a** C 1*s*, **b** N 1*s*, **c** O 1*s*, **d** Mn 2*p*, **e** Fe 2*p*, **f** Co 2*p*, **g** Ni 2*p*, and **h** Cu 2*p*.

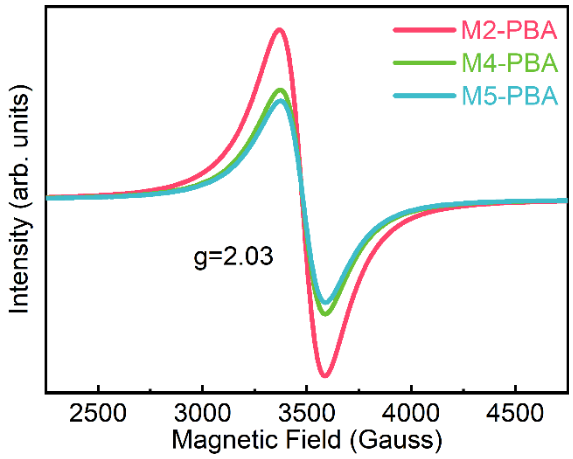

Fig. S9 EPR results of M2-PBA, M4-PBA and M5-PBA.

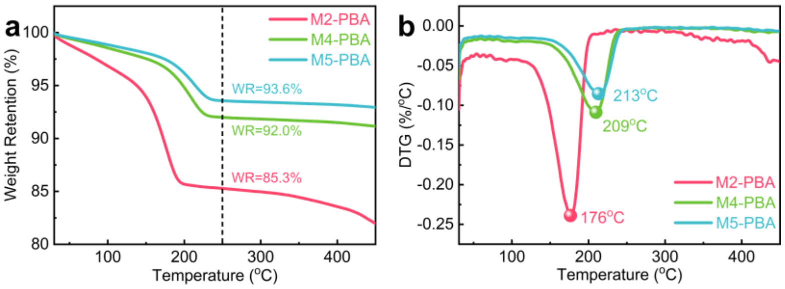

Fig. S10 **a** TGA, **b** DTG curves of M2-PBA, M4-PBA and M5-PBA.

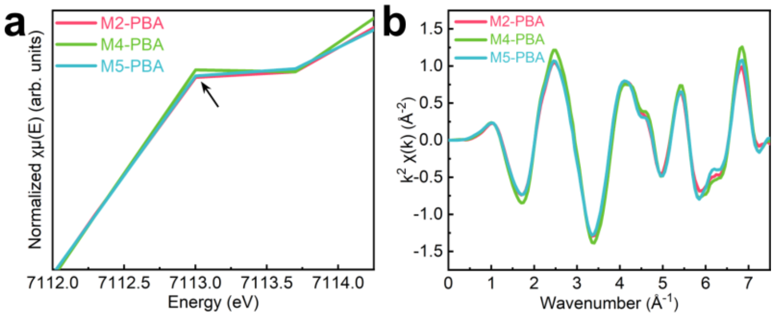

Fig. S11 XAFS of M2-PBA, M4-PBA and M5-PBA. **a** pre-edge for Fe K-edge, **b** spectra of EXAFS oscillations for Fe K-edge.

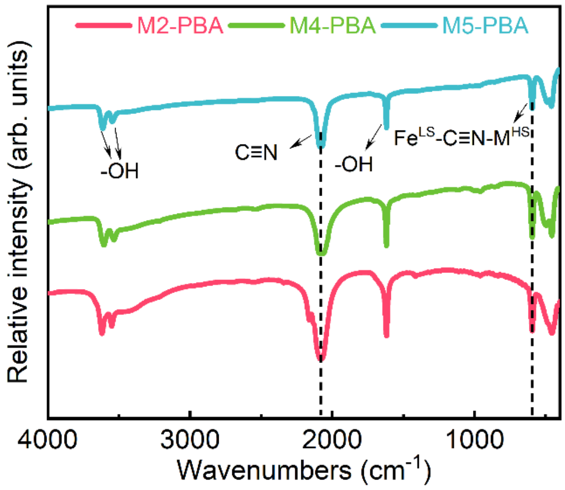

Fig. S12 FT-IR results of M2-PBA, M4-PBA and M5-PBA.

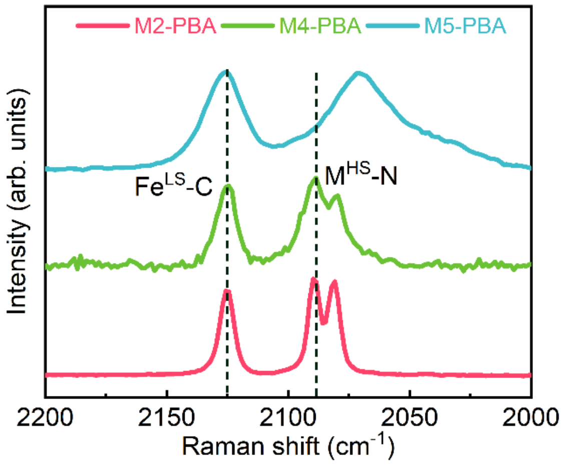

Fig. S13 Raman spectra of M2-PBA, M4-PBA and M5-PBA.

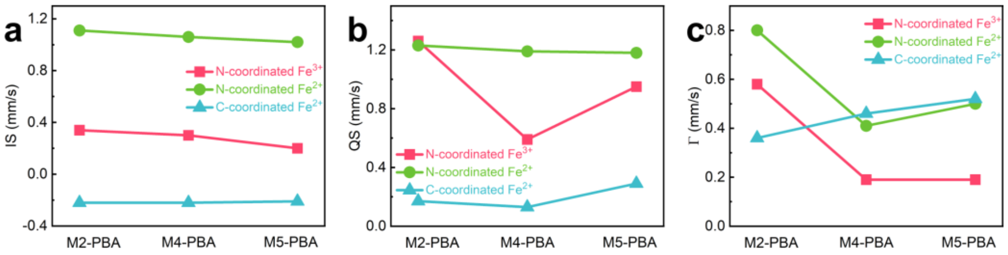

Fig. S14 **a** IS values, **b** QS values and **c** Γ comparation of Mössbauer spectra in M2-PBA, M4-PBA and M5-PBA.

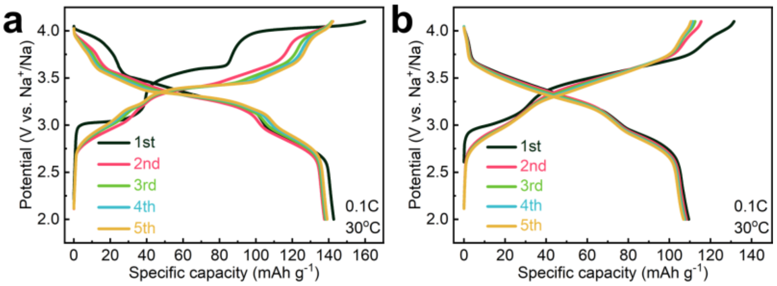

Fig. S15 The first five times galvanostatic charge and discharge curves of **a** M2-PBA and **b** M5-PBA at 30℃.

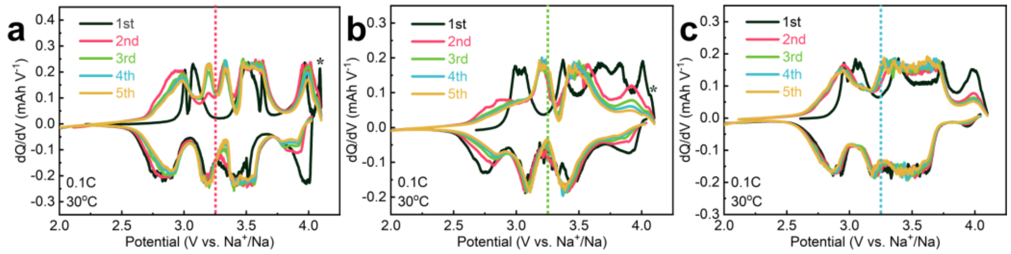

Fig. S16 Comparation of dQ/dV curves at 30°C. **a** M2-PBA, **b** M4-PBA, and **c** M5-PBA.

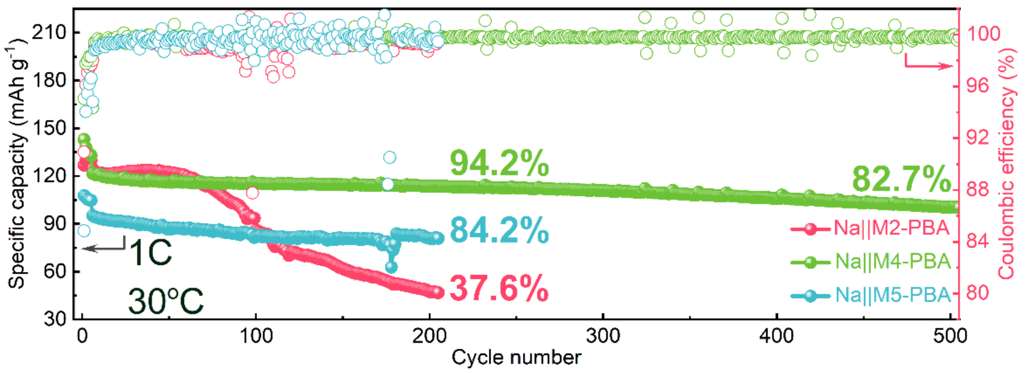

Fig. S17 Comparation of cycling performance at 1 C for M2-PBA, M4-PBA and M5-PBA at 30°C.

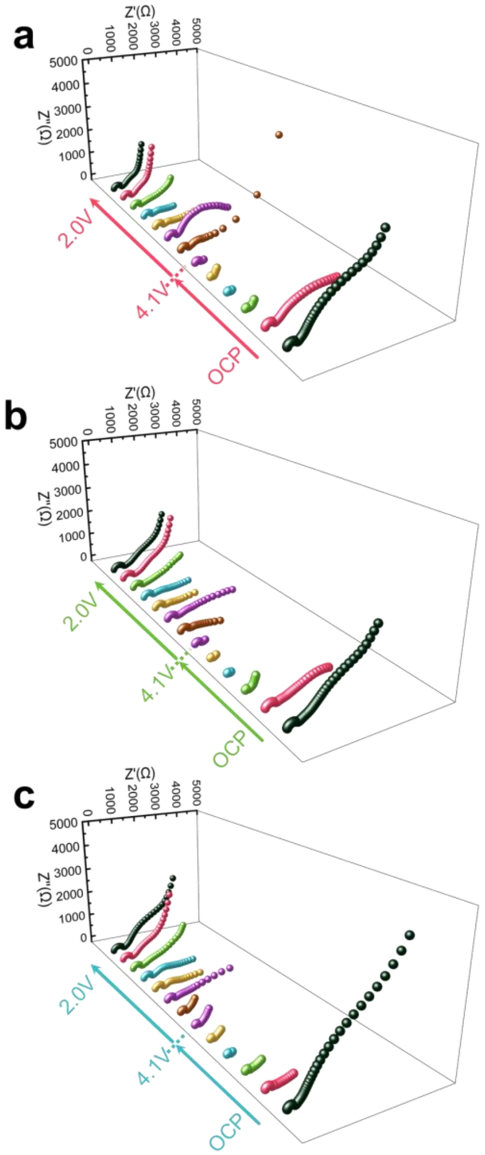

Fig. S18 Comparation of EIS testing results. **a** M2-PBA, **b** M4-PBA, and **c** M5-PBA.

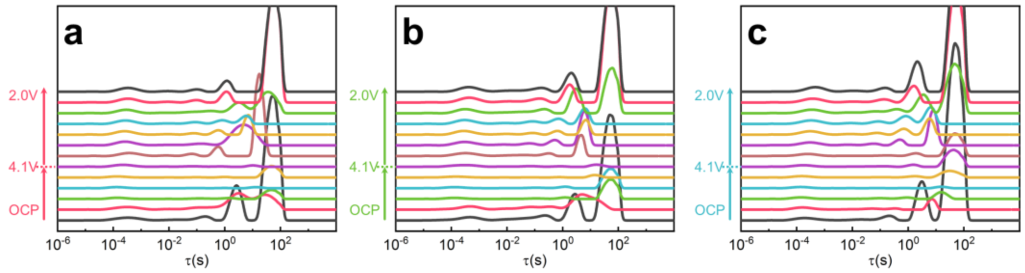

Fig. S19 Comparation of DRT results. **a** M2-PBA, **b** M4-PBA, and **c** M5-PBA.

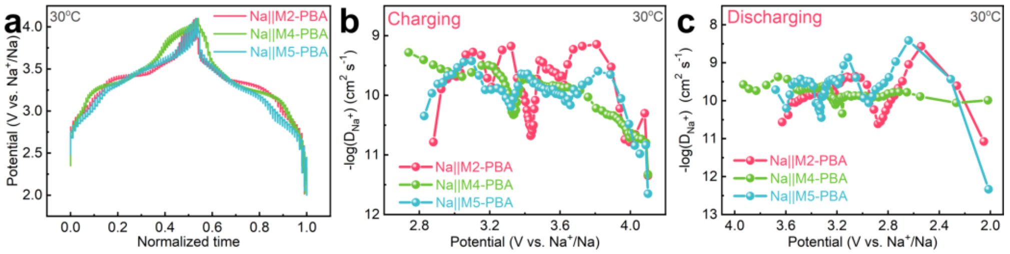

Fig. S20 **a** GITT testing curves, Na^+ diffusion coefficients altered with electrode voltage for **b** charging and **c** discharging procedure in M2-PBA, M4-PBA and M5-PBA at 30°C.

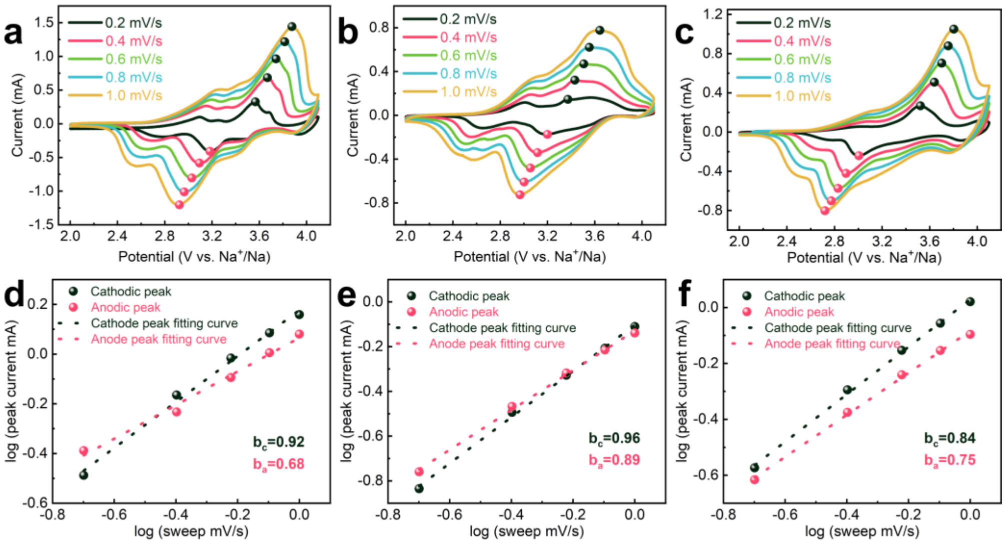

Fig. S21 CV curves at various scan rates: **a** M2-PBA, **b** M4-PBA, and **c** M5-PBA. Corresponding line-fitting slopes: **d** M2-PBA, **e** M4-PBA, and **f** M5-PBA.

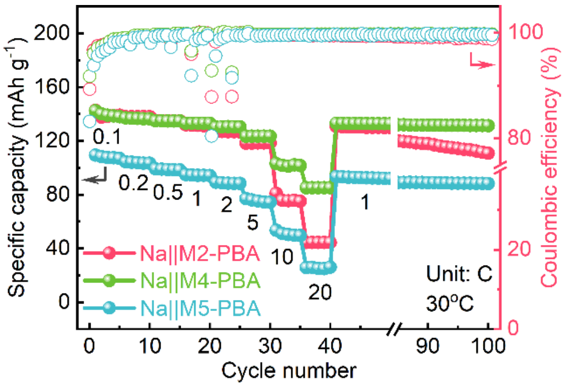

Fig. S22 Comparation of rate performance at 30°C.

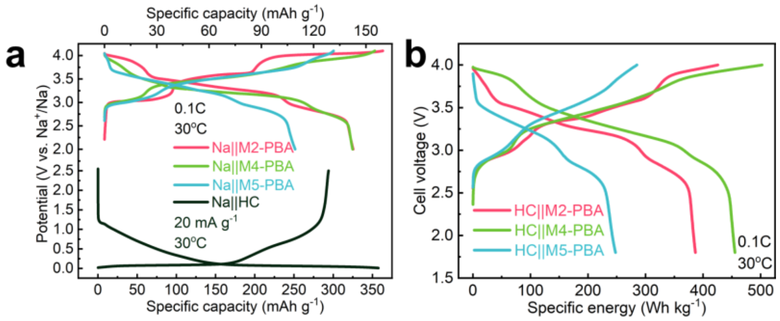

Fig. S23 Comparation of full cell electrochemical performance at 30°C. **a** specific capacity of PBA cathodes and hard carbon anode, **b** specific energy.

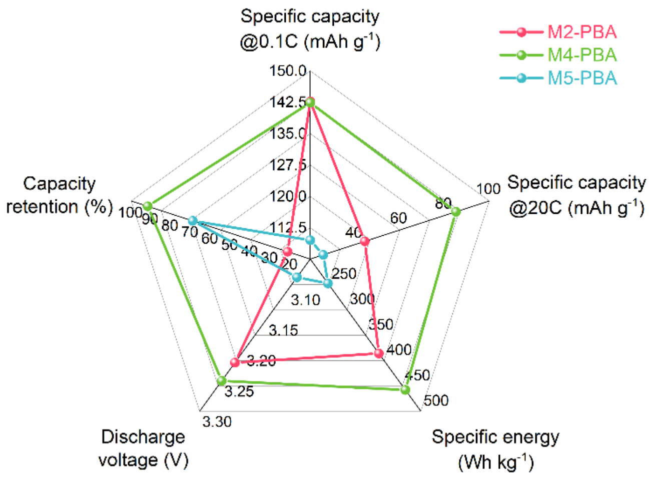

Fig. S24 Radar chart of electrochemical performance comparation for PBAs in this work.

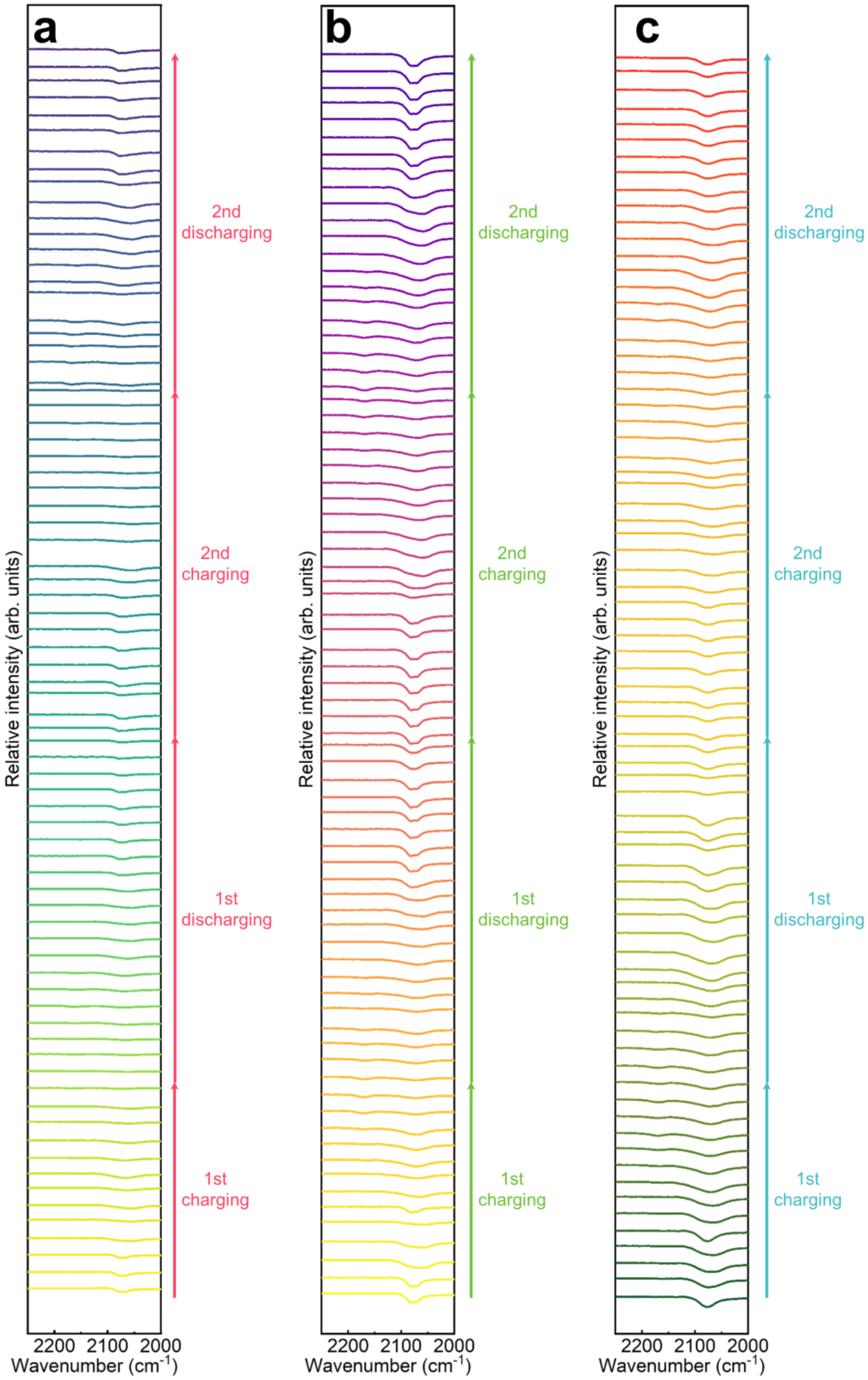

77

78 Fig. S25 Comparation of in-situ FT-IR full spectra during the first two cycles. **a** M2-PBA, **b** M4-

79 PBA, and **c** M5-PBA.

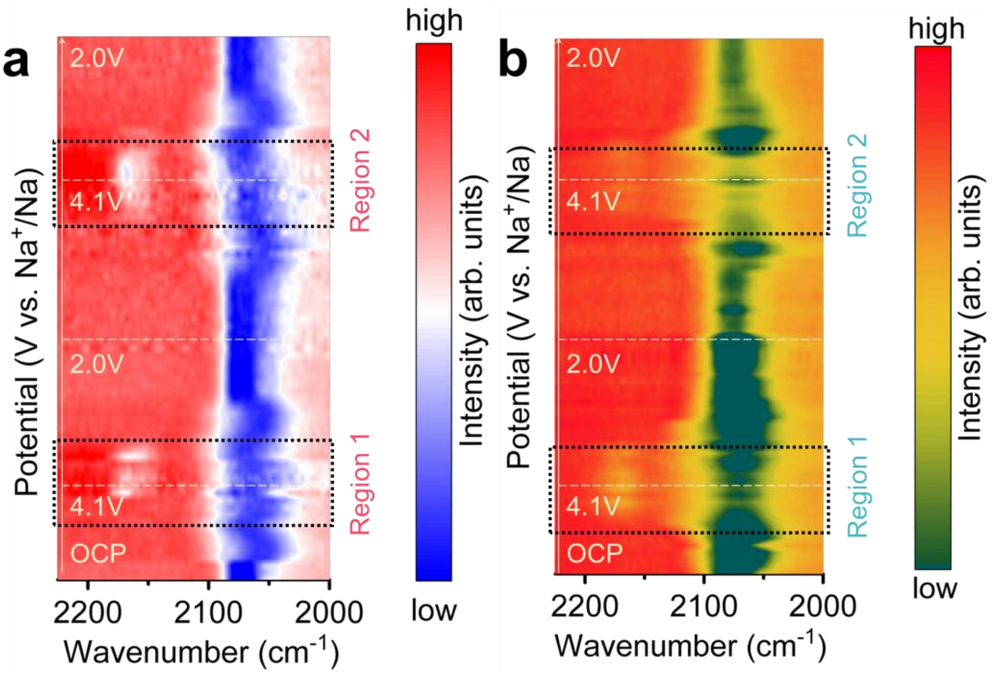

Fig. S26 In-situ FT-IR results of **a** M2-PBA, and **b** M5-PBA.

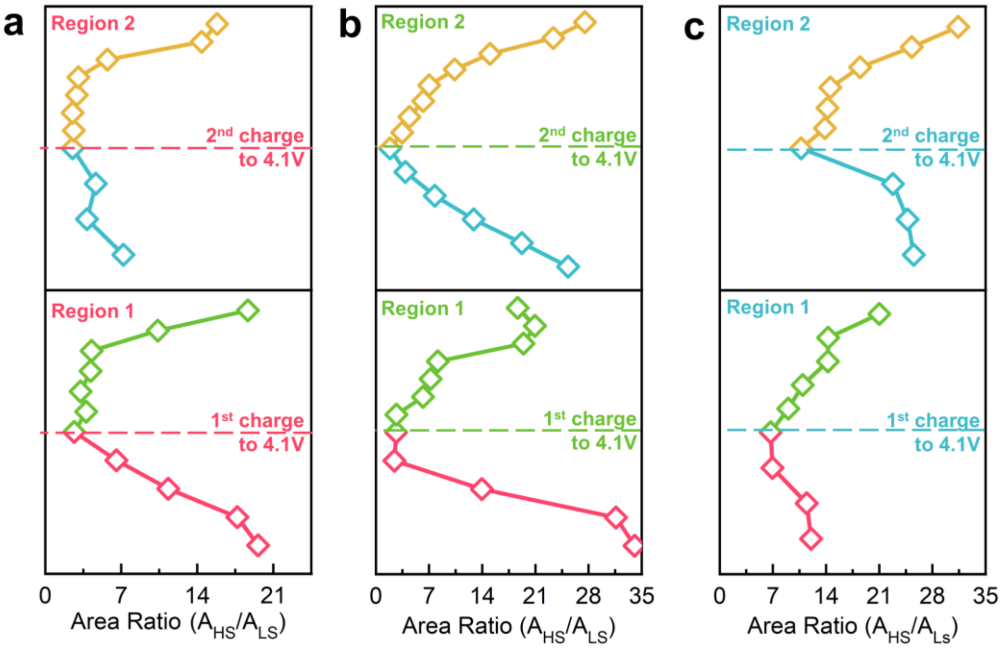

Fig. S27 The area ratios of peaks 1 and 2 in in-situ FT-IR for **a** M2-PBA, **b** M4-PBA and **c** M5-PBA.

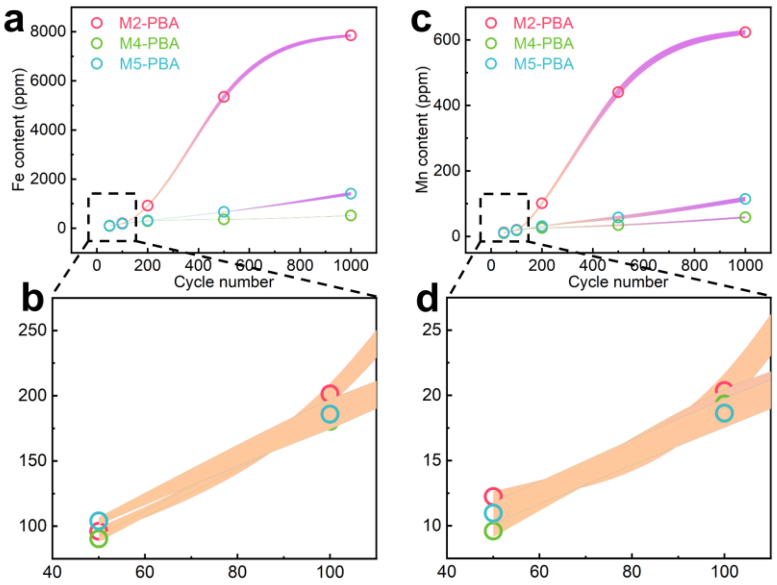

Fig. S28 Ex-situ ICP-OES testing results of the dissolvement metal elements in the electrolyte for M2-PBA, M4-PBA and M5-PBA with current density of 5C. Three sets of parallel samples form error bands, and the hollow circle mark represents the average of the three samples. **a**, **b** Fe content, and **c**, **d** Mn content.

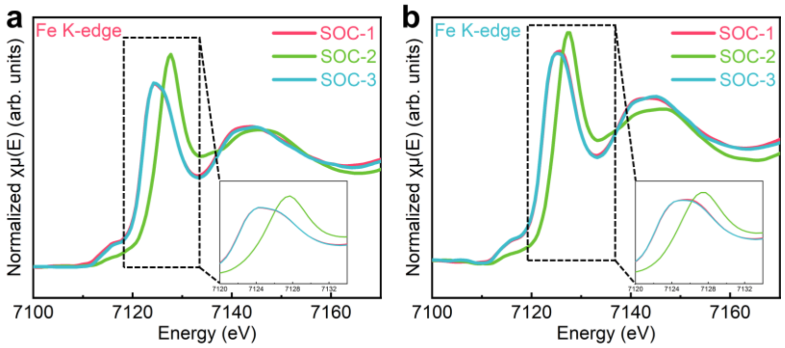

Fig. S29 Ex-situ XANES for Fe K-edge of **a** M2-PBA and **b** M5-PBA at different SOC with pre-edge in the insets. (The final state of charge for the first discharge cycle was defined as SOC-1, while the charging and discharging final states of charge for the second cycle were defined as SOC-2 and SOC-3, respectively.)

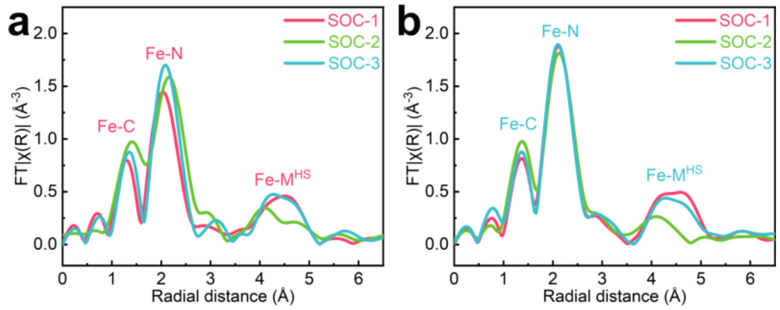

Fig. S30 Ex-situ EXAFS for Fe K-edge of **a** M2-PBA and **b** M5-PBA at different SOC. (The final state of charge for the first discharge cycle was defined as SOC-1, while the charging and discharging final states of charge for the second cycle were defined as SOC-2 and SOC-3, respectively.)

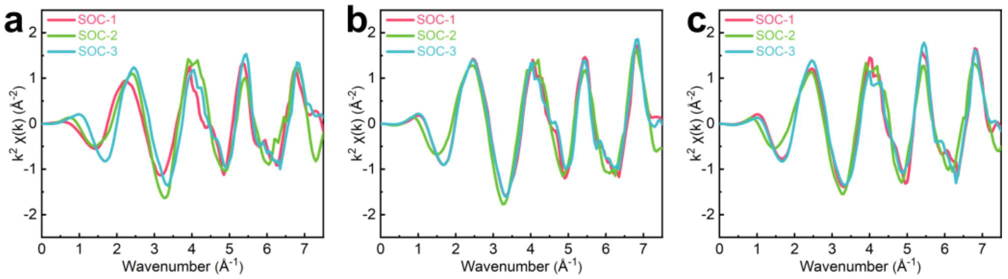

Fig. S31 Spectra of Fe K-edge EXAFS oscillations for **a** M2-PBA, **b** M4-PBA and **c** M5-PBA at different SOC. (The final state of charge for the first discharge cycle was defined as SOC-1, while the charging and discharging final states of charge for the second cycle were defined as SOC-2 and SOC-3, respectively.)

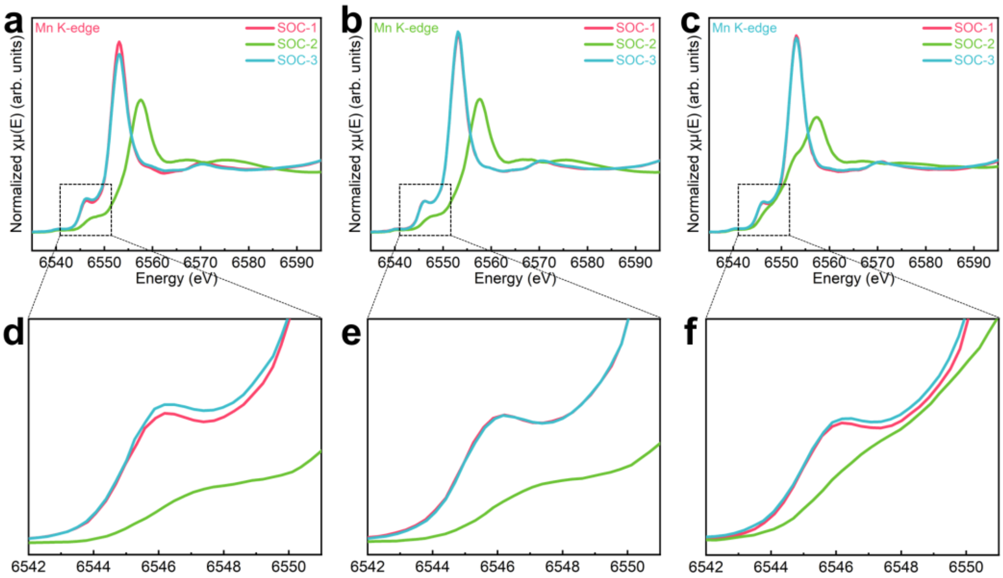

Fig. S32 Ex-situ XANES for Mn K-edge of **a** M2-PBA, **b** M4-PBA and **c** M5-PBA at different SOC with pre-edge **d** M2-PBA, **e** M4-PBA and **f** M5-PBA. (The final state of charge for the first discharge cycle was defined as SOC-1, while the charging and discharging final states of charge for the second cycle were defined as SOC-2 and SOC-3, respectively.)

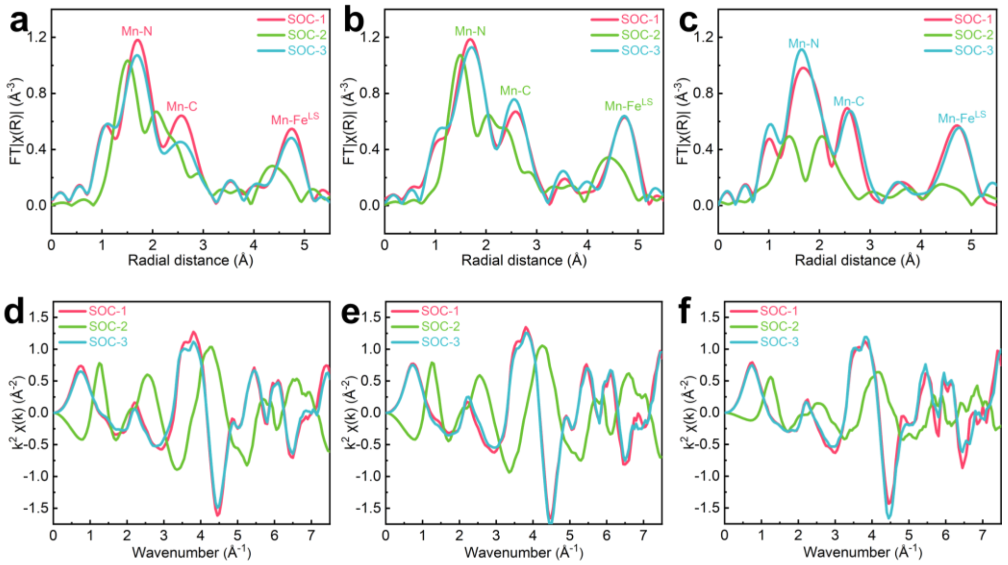

Fig. S33 Ex-situ FT-EXAFS for Mn K-edge of **a** M2-PBA, **b** M4-PBA and **c** M5-PBA with their spectra of EXAFS oscillations for **d** M2-PBA, **e** M4-PBA and **f** M5-PBA at different SOC. (The final state of charge for the first discharge cycle was defined as SOC-1, while the charging and discharging final states of charge for the second cycle were defined as SOC-2 and SOC-3, respectively.)

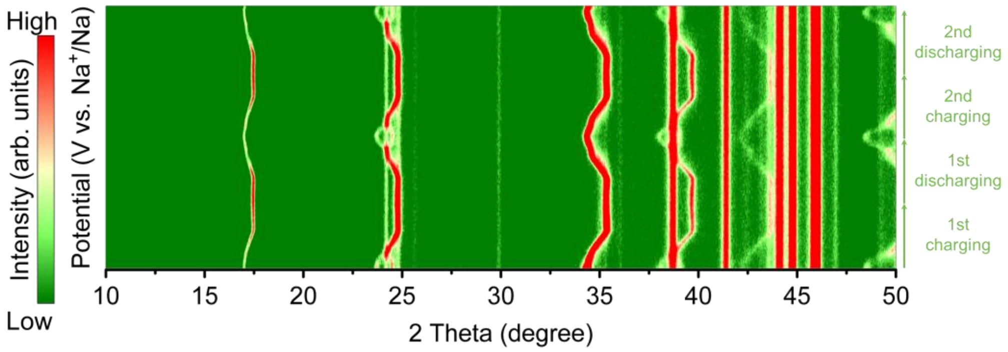

Fig. S34 In-situ XRD full patterns of M4-PBA.

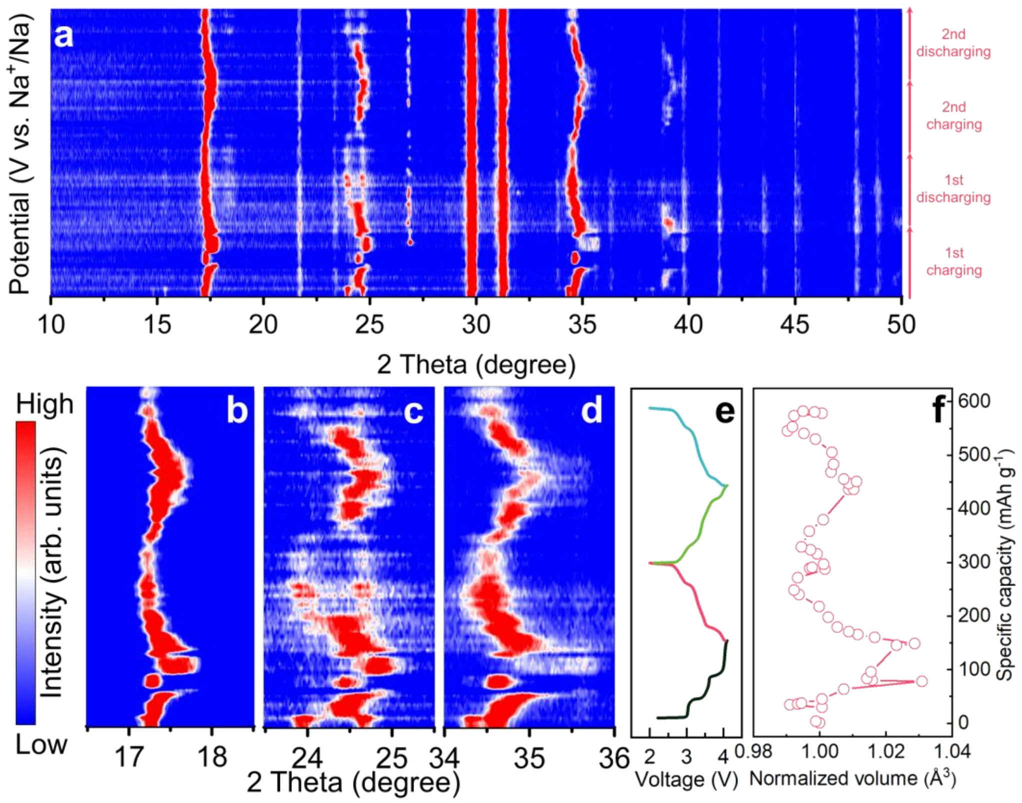

Fig. S35 Ex-situ XRD results of M2-PBA. **a** full pattern, **b-d** detailed patterns and corresponding **e** galvanostatic charge and discharge curves. **f** normalized unit cell volume change.

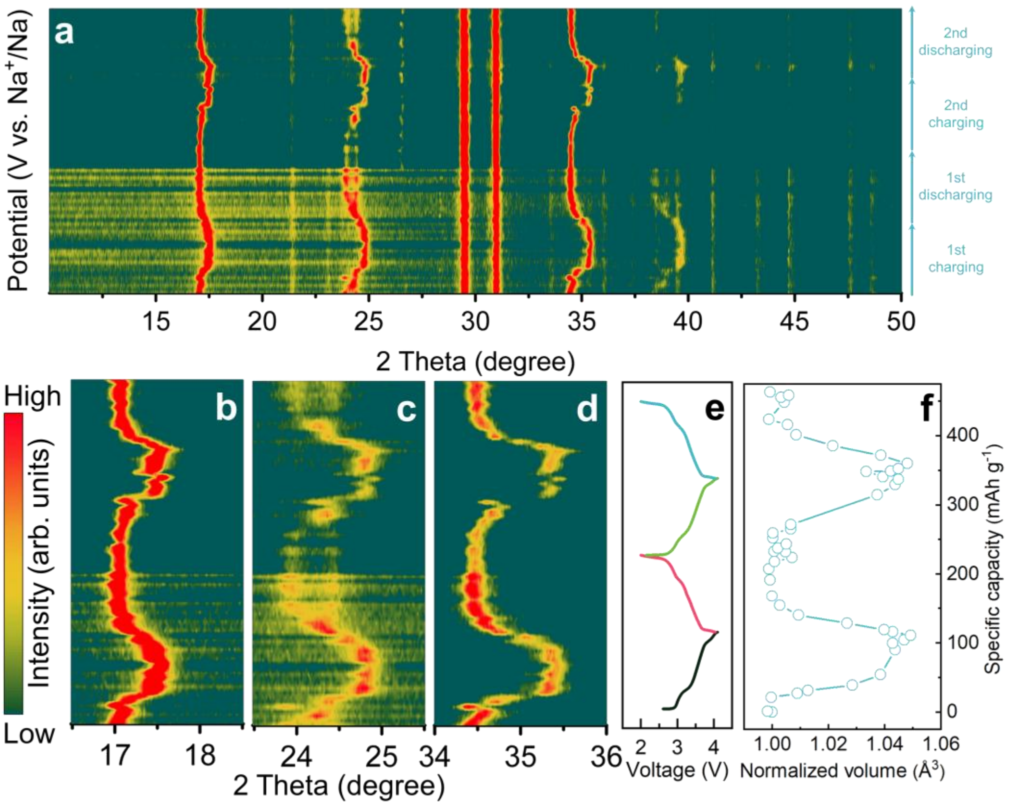

Fig. S36 Ex-situ XRD results of M5-PBA. **a** full pattern, **b-d** detailed patterns and corresponding **e** galvanostatic charge and discharge curves. **f** normalized unit cell volume change.

Table S1 The result of EA for PBAs

Sample	C (%)	N (%)	H (%)	O (%)
M2-PBA	17.48	20.39	0.90	7.15
M4-PBA	18.25	21.29	0.80	6.32
M5-PBA	19.07	22.24	0.65	5.19

Table S2 The result of ICP-OES for PBAs

Sample	Na (%)	Mn (%)	Fe (%)	Co (%)	Ni (%)	Cu (%)
M2-PBA	28.70	18.64	52.67	/	/	/
M4-PBA	28.73	9.81	43.57	9.74	8.15	/
M5-PBA	28.42	8.22	41.79	7.67	6.87	7.03

Table S3 Rietveld refined result of M2-PBA

M2-PBA Monoclinic, space group P21/n,

a = 10.54505 Å, b = 7.50288 Å, c = 7.27566 Å, V = 575.091 Å^3, $\alpha = \gamma$ = 90°, β = 92.498°

Atom	x	y	z	Occ.	Site
N1	0.0048	0.1959	0.1950	0.89	4e
N2	0.3005	0.5014	0.5101	0.89	4e
N3	0.0000	0.8081	0.2617	0.89	4e
C1	0.0050	0.3026	0.3130	0.89	4e
C2	0.1837	0.4998	0.5003	0.89	4e
C3	0.0037	0.6829	0.2998	0.89	4e
Na	0.2488	0.5154	0.0263	0.92	4e
Fe1	0.0000	0.5000	0.5000	0.50	2d
Mn	0.0000	0.0000	0.0000	0.50	2a
Fe2	0.0000	0.0000	0.0000	0.89	2a

Table S4 Rietveld refined result of M4-PBA

M4-PBA Monoclinic, space group *P*21/n,

a = 10.49118 Å, b = 7.48657 Å, c = 7.24433 Å, V = 568.430 Å^3, $\alpha = \gamma$ = 90°, β = 92.545°

Atom	x	y	z	Occ.	Site
N1	0.0048	0.1988	0.1967	0.91	4e
N2	0.3019	0.5042	0.5015	0.91	4e
N3	0.0033	0.8060	0.2094	0.91	4e
C1	0.0054	0.3036	0.3231	0.91	4e
C2	0.1833	0.5179	0.5169	0.91	4e
C3	0.0032	0.6839	0.3007	0.91	4e
Na	0.2289	0.4991	0.0236	0.945	4e
Fe1	0.0000	0.5000	0.5000	0.91	2d
Mn	0.0000	0.0000	0.0000	0.27	2a
Fe2	0.0000	0.0000	0.0000	0.27	2a
Co	0.0000	0.0000	0.0000	0.25	2a
Ni	0.0000	0.0000	0.0000	0.21	2a

Table S5 Rietveld refined result of M5-PBA

M5-PBA Monoclinic, space group *P*21/n,

a = 10.3043 Å, b = 7.4024 Å, c =7.1707 Å, V = 546.435 Å^3, $\alpha = \gamma$ = 90°, β = 92.501°

Atom	x	y	z	Occ.	Site
N1	0.0053	0.1997	0.1958	0.93	4e
N2	0.3011	0.5043	0.5026	0.93	4e
N3	0.0029	0.8054	0.2068	0.93	4e
C1	0.0062	0.3035	0.3244	0.93	4e
C2	0.1865	0.5271	0.5296	0.93	4e
C3	0.0034	0.6840	0.3001	0.93	4e
Na	0.2621	0.5389	0.0053	0.95	4e
Fe1	0.0000	0.5000	0.5000	0.93	2d
Mn	0.0000	0.0000	0.0000	0.23	2a
Fe2	0.0000	0.0000	0.0000	0.22	2a
Co	0.0000	0.0000	0.0000	0.20	2a
Ni	0.0000	0.0000	0.0000	0.18	2a
Cu	0.0000	0.0000	0.0000	0.17	2a

Table S6 Detailed fitting parameters of Mössbauer spectra

M2-PBA	IS (mm/s)	QS (mm/s)	Γ (mm/s)	Area (%)
N-coordinated Fe^{3+}	0.34	1.26	0.58	4.1
N-coordinated Fe^{2+}	1.11	1.23	0.8	28.9
C-coordinated Fe^{2+}	-0.22	0.17	0.46	67

M4-PBA	IS (mm/s)	QS (mm/s)	Γ (mm/s)	Area (%)
N-coordinated Fe^{3+}	0.3	0.59	0.19	2.9
N-coordinated Fe^{2+}	1.06	1.19	0.41	30
C-coordinated Fe^{2+}	-0.22	0.13	0.36	67.1

M5-PBA	IS (mm/s)	QS (mm/s)	Γ (mm/s)	Area (%)
N-coordinated Fe^{3+}	0.2	0.95	0.19	2.5
N-coordinated Fe^{2+}	1.02	1.18	0.5	28.2
C-coordinated Fe^{2+}	-0.21	0.29	0.52	69.2

Table S7 Comparison of the electrochemical performance of the as-prepared PBAs with other researcher for SIBs reported within the past three years

Samples	Capacity (mAh·g^{-1}@mA·g^{-1})	Cycle life (cycles, retention%@mA·g^{-1})	Rate performance (mAh·g^{-1}@ mA·g^{-1})	Reference
M4-PBA	142.4@14.875	1000, 91.7@743.75	85.1@2975	This work
MnNiPB-4xcit	93@100	500, 96@100	70@4000	*Angew. Chem. Int. Ed.*[1]
HE-PBA	120@10	100, 94@100	62@1000	*Adv. Mater.*[2]
R-PB	145@15	3500, 59.1@750	93.8@1500	*Adv. Energy Mater.*[3]
HEM-HCF	117@100	200, 90@100	79@1000	*Adv. Funct. Mater.*[4]
FeZn-PB	145@20	500, 74.9@1000	98.5@1000	*Chem. Eng. J.*[5]
SC-HEPBA	115.4@100	1000, 76.1@500	74.4@3000	*Energy Stor. Mater.*[6]
HE-HCF	105.1@15	10000, 95@1500	55.7@3000	*Angew. Chem. Int. Ed.*[7]
MnHCF-5%Co_xB	149@34	450, 81@750	130@1700	*Angew. Chem. Int. Ed.*[8]
NKPB-3	147.9@15	300, 83.5@150	about 105@300	*Angew. Chem. Int. Ed.*[9]
K-FeMnPBA3	139.1@100	700, 77.1@100	118.3@3000	*Adv. Funct. Mater.*[10]
PB-130	113.6@30	1200, 85.5@1200	86@2000	*ACS Nano*[11]
HE-PW	129@10	1000, about 67@500	73@500	*Angew. Chem. Int. Ed.*[12]
PBA(Cu)□Fe	135.7@20	200, 78.5@500	77.5@1000	*Adv. Funct. Mater.*[13]
MnCuNi-PBA	116@10	500, 81.8@100	66.2@2000	*Adv. Mater.*[14]
MNHCF-3	about 100@15	10000, 90.1@750	90.9@3000	*ACS Nano*[15]
SP-PBA	120.6@25	1500, 85.7@500	80@2500	*Adv. Mater.*[16]
NaFeHCF@rGO	124.6@25	1000, 81@1000	102.4@4000	*Adv. Energy Mater.*[17]
EDTA-1MVC	122.5@100	10000, 86.32@6000	82@6000	*Small*[18]
PB-325	131@24	NA	99@1200	*Chem. Eng. J.*[19]
AgHCF@CNTs	168.4@50	500, 74@500	90.7@2000	*ACS Energy Lett.*[20]
HC-PB	140@34	1000, 94.6@1700	105@5100	*Energy Stor. Mater.*[21]

Supplementary References

1 Gebert, F. et al. Epitaxial nickel ferrocyanide stabilizes Jahn-Teller distortions of manganese ferrocyanide for sodium-ion batteries. *Angew. Chem. Int. Ed. Engl.* **60**, 18519-18526 (2021).

2 Ma, Y. et al. High-entropy metal-organic frameworks for highly reversible sodium storage. *Adv. Mater.* **33**, e2101342 (2021).

3 Peng, J. et al. Processing rusty metals into versatile prussian blue for sustainable energy storage. *Adv. Energy Mater.* **11**, 2102356 (2021).

4 Ma, Y. et al. Resolving the role of configurational entropy in improving cycling performance of multicomponent hexacyanoferrate cathodes for sodium-ion batteries. *Adv. Funct. Mater.* **32**, 2202372 (2022).

5 Zhang, L. L. et al. Effect of Zn-substitution induced structural regulation on sodium storage performance of Fe-based Prussian blue. *Chem. Eng. J.* **433**, 133739 (2022).

6 Huang, Y. et al. Boosting the sodium storage performance of Prussian blue analogs by single-crystal and high-entropy approach. *Energy Stor. Mater.* **58**, 1-8 (2023).

7 Peng, J. et al. A disordered rubik's cube-inspired framework for sodium-ion batteries with ultralong cycle lifespan. *Angew. Chem. Int. Ed. Engl.* **62**, e202215865 (2023).

8 Xu, C. et al. Surface engineering stabilizes rhombohedral sodium manganese hexacyanoferrates for high-energy Na-ion batteries. *Angew. Chem. Int. Ed. Engl.* **62**, e202217761 (2023).

9 Zhang, H. et al. Prussian blue analogues with optimized crystal plane orientation and low crystal defects toward 450 Wh kg^{-1} alkali-ion batteries. *Angew. Chem. Int. Ed. Engl.* **62**, e202303953, (2023).

10 Gao, Y. et al. Structurally stable, low H_2O Prussian blue analogs toward high performance sodium storage. *Adv. Funct. Mater.* **34**, 2314860 (2024).

11 Ge, L. et al. Elaborating the crystal water of Prussian blue for outstanding performance of sodium ion batteries. *ACS Nano* **18**, 3542-3552 (2024).

12 He, Y. et al. Entropy-mediated stable structural evolution of Prussian white cathodes for long-life Na-ion batteries. *Angew. Chem. Int. Ed. Engl.* **63**, e202315371 (2024).

13 Liu, J. C. et al. Boosting sodium storage in Prussian blue analogs through iron vacancies and copper doping. *Adv. Funct. Mater.* **34**, 2314167 (2024).

14 Luo, Y. et al. Inhibiting the Jahn-Teller effect of manganese hexacyanoferrate via Ni and Cu codoping for advanced sodium-ion batteries. *Adv. Mater.* **36**, e2405458 (2024).

15 Peng, J. et al. Structural engineering of Prussian blue analogues enabling all-climate and ultralong cycling sodium-ion batteries. *ACS Nano*, **18**, 19854-19864 (2024).

16 Tang, C. et al. Toward ultrahigh rate and cycling performance of cathode materials of sodium ion battery by introducing a bicontinuous porous structure. *Adv. Mater.* **36**, e2402005 (2024).

17 Tang, Y. et al. Epitaxial nucleation of $Na_xFeFe(CN)_6$@rGO with improved lattice regularity as ultrahigh-rate cathode for sodium-ion batteries. *Adv. Energy Mater.* **14**, 202303015 (2024).

18 Wang, Y. et al. Highly crystalline multivariate Prussian blue analogs via equilibrium chelation strategy for stable and fast charging sodium-ion batteries. *Small*, e2403211 (2024).

19 Wang, Z. et al. Charge transfer induced highly active low-spin iron of Prussian blue cathode through calcination strategy for high performance sodium-ion batteries. *Chem. Eng. J.* **488**, 151090 (2024).

20 Zhao, X. Y. et al. Four-electron redox reaction in Prussian blue analogue cathode material for high-rerformance sodium-ion batteries. *ACS Energy Lett.* **9**, 2748-2757 (2024).

21 Ma, H. et al. Medium-mediated high-crystalline Prussian blue toward exceptionally boosted sodium energy storage. *Energy Stor. Mater.* **70**, 103411 (2024).
