# Supplementary material for: Tuning cyanide coordination electronic structure enables stable Prussian blue analogues for sodium-ion batteries
Source: Nat Commun. 2025 Nov 18;16:10083. doi: 10.1038/s41467-025-65062-x (PMC12627476; doi:10.1038/s41467-025-65062-x)
Supplement: Supplementary file 2 — Description of Additional Supplementary File [file 41467_2025_65062_MOESM2_ESM.pdf]

Description of Additional Supplementary Files

Title: Supplementary Data 01
Description: The atomic coordinates of cubic phase Mn-PBA in Fig 1a

Title: Supplementary Data 02
Description: The atomic coordinates of cubic phase Fe-PBA in Fig 1b

Title: Supplementary Data 03
Description: The atomic coordinates of cubic phase Co-PBA in Fig 1c

Title: Supplementary Data 04
Description: The atomic coordinates of cubic phase Ni-PBA in Fig 1d

Title: Supplementary Data 05
Description: The atomic coordinates of cubic phase Cu-PBA in Fig 1e

Title: Supplementary Data 06
Description: The atomic coordinates of rhombohedral phase Mn-PBA in Fig 1f

Title: Supplementary Data 07
Description: The atomic coordinates of rhombohedral phase Fe-PBA in Fig 1g

Title: Supplementary Data 08
Description: The atomic coordinates of rhombohedral phase Co-PBA in Fig 1h

Title: Supplementary Data 09
Description: The atomic coordinates of rhombohedral phase Ni-PBA in Fig 1i

Title: Supplementary Data 10
Description: The atomic coordinates of rhombohedral phase Cu-PBA in Fig 1j

Title: Supplementary Data 11
Description: The atomic coordinates of M2-PBA in Fig 5d

Title: Supplementary Data 12
Description: The atomic coordinates of M4-PBA in Fig 5e

Title: Supplementary Data 13
Description: The atomic coordinates of M5-PBA in Fig 5f
